# Pneumatosis Intestinalis: To Biopsy or Not to Biopsy?

**DOI:** 10.7759/cureus.12140

**Published:** 2020-12-18

**Authors:** Karen Medgyesy, Radiana Trifonova, Travis Bevington, Micheal Tadros

**Affiliations:** 1 Gastroenterology, Albany Medical Center, Albany, USA; 2 Pathology, Albany Medical Center, Albany, USA; 3 Radiology, Albany Medical Center, Albany, USA; 4 Gastroenterology and Hepatology, Albany Medical Center, Albany, USA

**Keywords:** pneumatosis intestinalis, pneumatosis cystoides intestinalis, screening colonoscopy

## Abstract

Pneumatosis intestinalis (PI) is a rare condition characterized by multiple air-filled cystic lesions in the submucosa or subserosa of the intestine. Despite a limited understanding of its pathogenesis, the causes of PI can be categorized into life-threatening or benign, which helps guide patient management. For benign etiologies, interventions should be minimized and endoscopic maneuvers should be avoided as most of these cases can be managed conservatively. We present a patient with asymptomatic, benign PI who subsequently developed symptoms following cyst biopsy during a screening colonoscopy.

## Introduction

Pneumatosis intestinalis (PI) is an uncommon condition characterized by multiple air-filled cystic lesions in the submucosa or subserosa of the intestine. Patient presentation ranges from asymptomatic to diarrhea, abdominal pain, constipation, or rectal bleeding [1]. PI itself is not a disease, but a radiographic or physical finding [2].

Primary, or idiopathic, PI accounts for 15% of cases. It is usually asymptomatic and predominantly located in the colon. Secondary PI makes up the remaining 85%. It is associated with concomitant respiratory system or gastrointestinal tract disorders as well as immunodeficiency, bacterial infection, viral infection, and certain drugs [3].

Causes of PI can be categorized as life-threatening or benign. Life-threatening etiologies include intestinal ischemia, mesenteric vascular disease, intestinal obstruction, enteritis, colitis, ingestion of corrosive agents, toxic megacolon, and collagen vascular disease. Benign causes outnumber life-threatening causes and are primary PI, pulmonary diseases, other intestinal conditions, iatrogenic causes, and certain medications [4]. In patients in the benign category, interventions need to be minimized and endoscopic maneuvers should be avoided. 

## Case presentation

**Figure 1:**
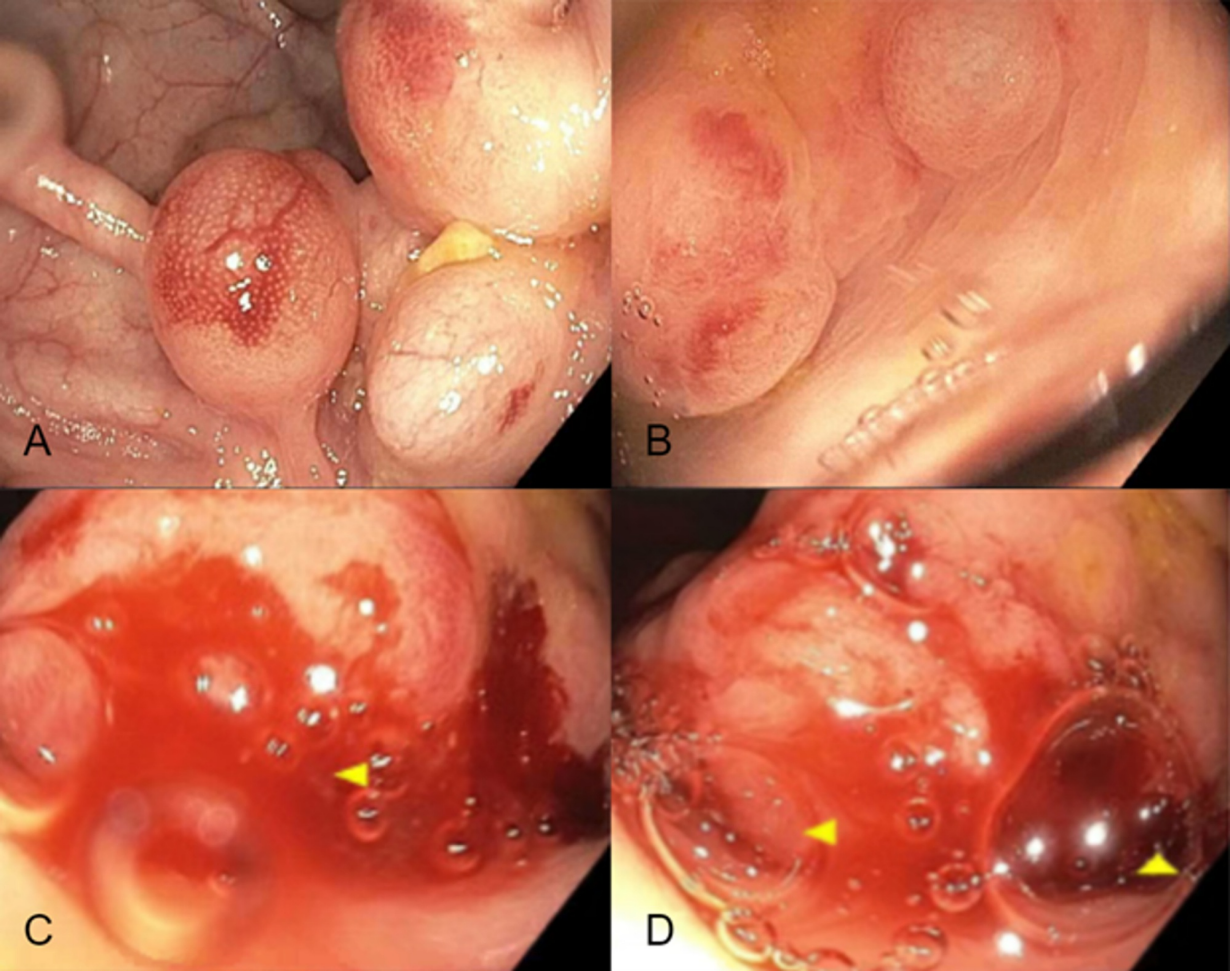
Colonoscopy (A-B) Polypoid cysts with red overlying mucosa in the proximal sigmoid and distal descending colon. (C-D) Deflation of a 10mm polypoid lesion of the proximal sigmoid colon with cystic air bubbles.

A 51-year-old male with a past medical history of hypothyroidism presented for average-risk screening colonoscopy. The patient’s history was negative for smoking and alcohol consumption. During the colonoscopy, multiple polypoid cysts with red overlying mucosa were found in the proximal sigmoid and distal descending colon (Figure 1A-1B). Cystic air emerged with the biopsy of a benign 10 mm polypoid lesion in the proximal sigmoid colon (Figure 1C-1D). The patient was discharged with instructions for a computed tomography (CT) abdomen and pelvis with contrast at his next follow-up appointment.

Five days later the patient presented to the Emergency Department due to severe hiccups to the point of vomiting to obtain relief as well as constipation with only one small bowel movement since the colonoscopy. Aside from a borderline heart rate of 100 bpm, vitals were stable. His abdomen was soft and nondistended with mild diffuse tenderness to palpation. Bowel sounds were normal. He had no rebound, guarding, or organomegaly. A CT of the abdomen and pelvis with contrast showed no evidence of pneumatosis intestinalis or free air, though it was limited by a region of streak artifact of the mid-abdomen.

The patient was admitted for observation due to his symptoms and previous colonoscopy findings. His lab results were a normal white blood count of 8.7 x 10^3^/uL, decreased potassium of 3.3 mEq/L, normal creatinine of 1.1 mg/dL, normal bilirubin of 1.0 mg/dL, normal transaminases (aspartate transaminase of 25 IU/L and alanine transaminase of 17 IU/L), and normal blood urea nitrogen of 13 mg/dL. A bowel regimen of docusate, senna, and milk of magnesia was used to alleviate his constipation. Due to marked improvement of his symptoms with chlorpromazine and ondansetron, he was discharged the next day with follow-up instructions.

**Figure 2:**
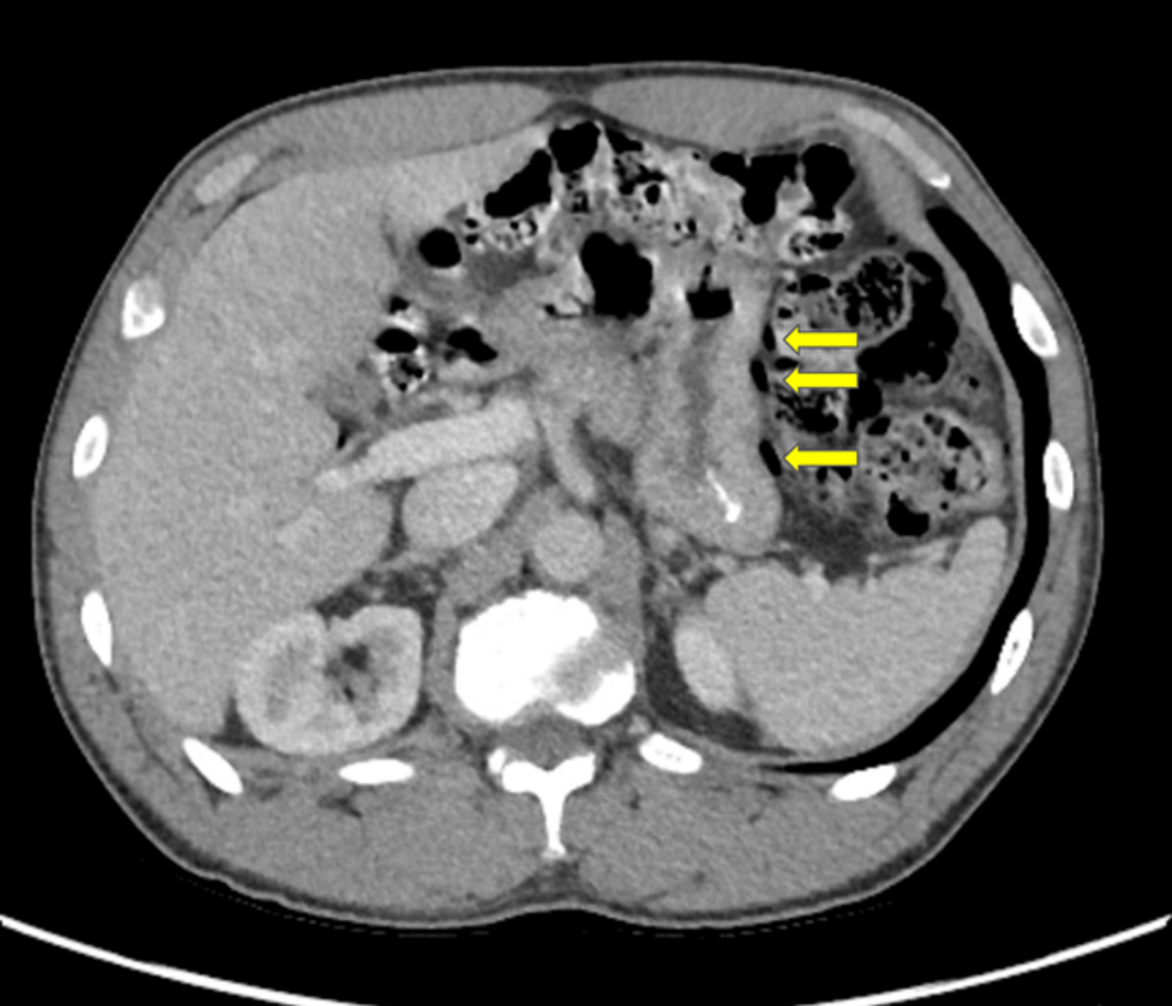
Follow-up CT of the abdomen and pelvis with contrast Computed tomography (CT) with curvilinear foci of air in the wall of the large bowel at the splenic flexure without bowel wall thickening.

A follow-up CT of the abdomen and pelvis with contrast approximately five weeks after discharge showed curvilinear foci of air in the large bowel at the splenic flexure and less conspicuously along the proximal transverse and right colon (Figure 2). The bowel wall did not show thickening, which is more indicative of benign PI (Figure 2).

**Figure 3:**
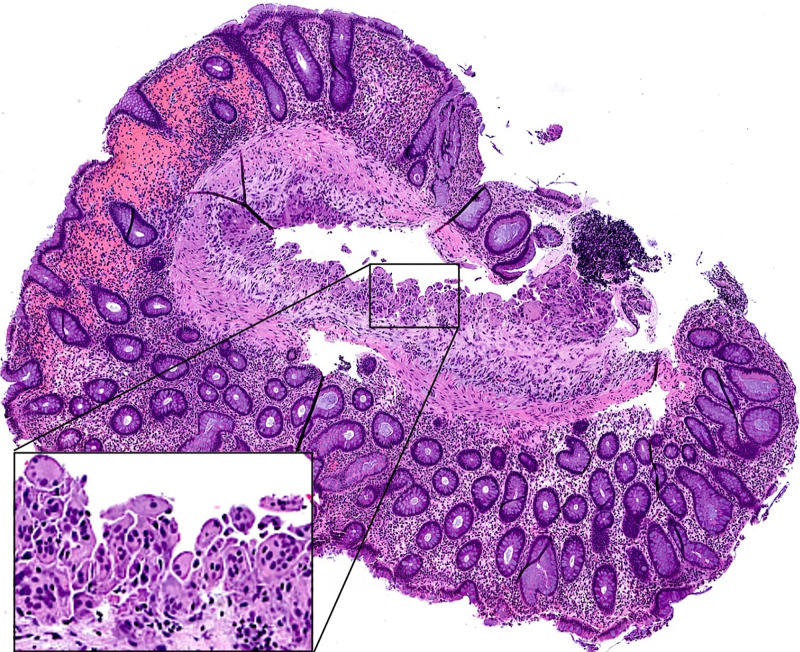
Microscopic image of sigmoid colon biopsy Hematoxylin and eosin (H&E) staining, original magnification x400. Inset shows the cyst lining with histiocytes and multinucleated giant cells.

Pathology of the sigmoid colon polyp revealed a submucosal cyst lined by histiocytes and multinucleated giant cells, consistent with PI (Figure 3).

## Discussion

PI is estimated to affect 0.03% of the population and is being detected more frequently due to increased CT scanning [4]. Despite first being described in French literature in 1754 by Duvernoy [4], it is still poorly understood. A few theories of its pathogenesis have been proposed [5]. In the mechanical theory, gas enters the bowel wall from the intestinal lumen in times of increased intraluminal pressure; for example, when there is intestinal obstruction due to a gastrointestinal tumor. In the pulmonary theory, gas from the lung reaches the intestinal wall through the mediastinum. The alveoli may rupture in chronic obstructive pulmonary disease, which causes pneumomediastinum that dissects into the aorta and mesenteric vessels that terminate in the bowel wall. The bacterial theory involves gas-forming bacteria invading the submucosa in times of increased mucosal permeability and producing gas in the intestinal wall. Similarly, the chemical and nutritional theory involves increased gas production due to bacterial fermentation of carbohydrates, which penetrates the intestinal wall [5]. Inflammation, physical damage of the intestinal mucosa, gastrointestinal dysmotility, and immune dysfunction may play a role as well [6].

Although PI is rare, benign cases should be recognized, and biopsy should be avoided to prevent complications. Systemic analysis of 239 PI cases found the efficacy of conservative treatment reached 93.3% [7]. This is especially important considering the growing number of colonoscopies performed worldwide [8]. Some authors recommend a biopsy to confirm diagnosis, as PI lesions can appear similar to inflammatory bowel disease, neoplasms, and polyps [2,9], but this case shows that there is risk associated with biopsy. Additionally, the removal of a polyp that was actually a PI cyst may cause bowel perforation [2]. The rate of bowel perforation rate during colonoscopy is small, ranging from 0.005-0.0085%, but is the most frequent colonoscopy complication resulting in mortality [8].

Complications could have been avoided in our patient as he was asymptomatic prior to the colonoscopy. His lab findings were also not concerning for pathologic PI. A retrospective multicenter study of 500 PI patients found a significant association of elevated white blood count, potassium, creatinine, bilirubin, transaminases, and blood urea nitrogen greater than 20 mg/dL with pathologic PI [10]. These results were normal apart from decreased potassium.

Endoscopists should familiarize themselves with the endoscopic appearance of PI and, if biopsied, should consider defect closure with clips. The description of PI cysts varies in the literature. One study notes two characteristic appearances and that the cysts usually collapse with biopsy. They are either larger cysts, up to 3 cm, with red overlying mucosa or a group of small white cysts atop sub-atrophic mucosa [11]. Other studies describe the submucosal cysts as polypoid with a bluish overlying mucosa while the subserosal cysts also have a bluish hue, but are a group of gas-filled blebs [12,13]. Familiarity with these patterns can help endoscopists recognize benign PI and determine the need for biopsy. 

## Conclusions

Despite its rarity, it is important for clinicians, especially endoscopists, to recognize PI. Benign PI can be treated conservatively and should not be biopsied during colonoscopy. A biopsy can cause complications, including life-threatening bowel perforation, so it is important to raise awareness among endoscopists to recognize this finding. If biopsied, clipping can be considered to close the defect.
